# Supplementary material for: Functionalization of Photosensitized Silica Nanoparticles for Advanced Photodynamic Therapy of Cancer
Source: Int J Mol Sci. 2021 Jun 21;22(12):6618. doi: 10.3390/ijms22126618 (PMC8234454; doi:10.3390/ijms22126618)
Supplement: Supplementary file 1 [file ijms-22-06618-s001.zip › ijms-1258421-supplementary.pdf]

ESI for:

Functionalization of photosensitized silica nanoparticles for advanced photodynamic therapy for cancer

Ruth Prieto-Montero[1§], Alejandro Prieto-Castañeda[2§], Alberto Katsumiti[3,4§], Miren P. Cajaraville[3], Antonia R. Agarrabeitia[2], María J. Ortiz[2*], Virginia Martínez-Martínez[1*].

1 Departamento de Química Física, Universidad del País Vasco/Euskal Herriko Unibertsitatea (UPV/EHU), 48080 Bilbao, Spain

2 Departamento de Química Orgánica, Facultad de CC. Químicas, Universidad Complutense de Madrid, 28040 Madrid, Spain

3 CBET Research Group, Dept. Zoology and Animal Cell Biology; Faculty of Science and Technology and Research Centre for Experimental Marine Biology and Biotechnology PiE, University of the Basque Country UPV/EHU, 48620, Basque Country, Spain.

4 GAIKER Technology Centre, Basque Research and Technology Alliance (BRTA), 48170 Zamudio, Spain.

1. **SEM, XPS and FTIR** **S2**
2. **Absorption spectra and Photophysical data** **S3**
3. **MMT assays** **S4**
4. **EC50 values** **S5**
5. **Fluorescence image** **S6**
6. **Synthesis of new BODIPY-based PSs** **S7**
7. **Synthesis of MSNs** **S8**
8. **Functionalization of FA** **S9**
9. **Cell illumination** **S10**
10. **RMN spectra** **S11**
11. **References** **S16**

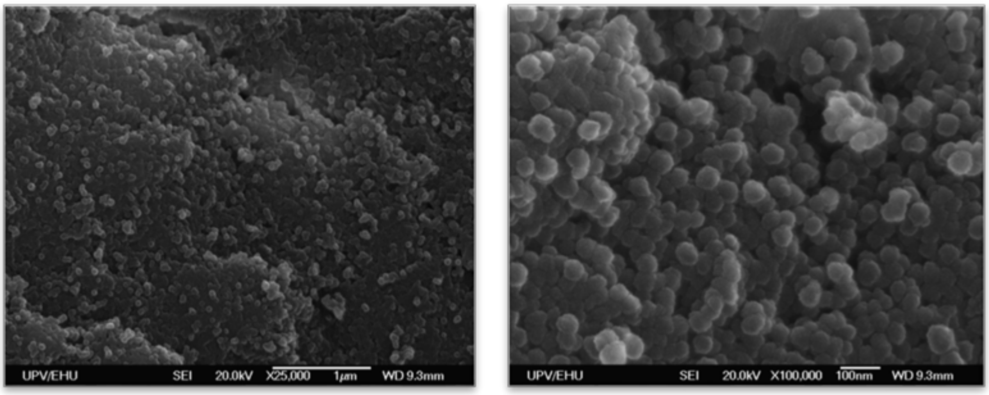

Figure S1: SEM image of MSNs.

Table S1. XPS data of mesoporous silica nanoparticles in water.

Name	Shell	XPS (% At rel)			
		C	O	Si	N
NH-MSN	NH_2/OH	23.6	46.0	25.4	5.0
CN-MSN	CN/OH	38.8	36.6	19.2	5.2
COOH-MSN	COOH/OH	25.5	50.4	24.1	-

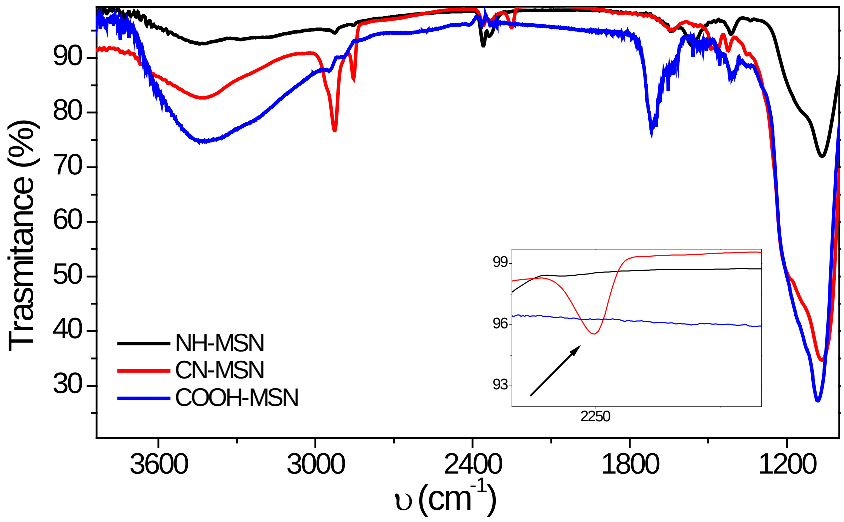

Figure S2. Infrared spectra of NH-MSN (black), CN-MSN (red) and COOH-MSN (blue) and from 2500 cm^{-1} to 2150 cm^{-1} (inset).

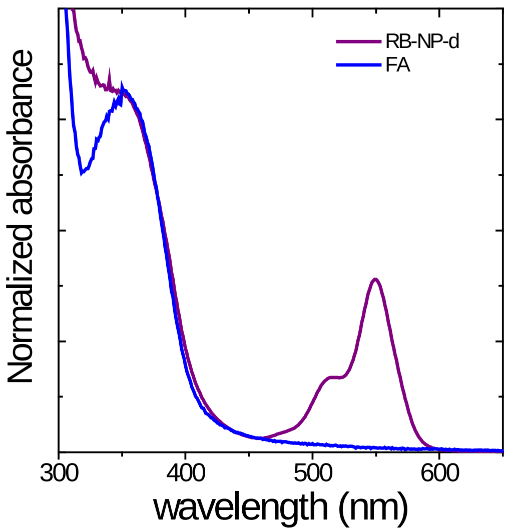

Figure S3. Normalized absorption spectra of folic acid (blue) and RB-PEG-NP-**d** (purple) in aqueous solution (0.5 mg/mL).

Table S2. Photophysical parameters and singlet oxygen quantum yields for graftable-PSs; absorption maxima (λ_{ab}), molar absorption coefficient (ε_{max}), fluorescence maxima (λ_{fl}), fluorescence quantum yield (Φ_{fl}), fluorescence lifetime (τ_{fl}), singlet oxygen quantum yield (Φ_{Δ}) and Phototoxic Power (PP = ε x Φ_{Δ}).

	λ_{ab} (nm)	ε_{max} 10^{-4} (M^{-1} cm^{-1})	λ_{fl} (nm)	Φ_{fl}	τ_{fl} (ns)	Φ_{Δ}	PP 10^{-4} (M^{-1} cm^{-1})
BDP1	447.0	4.3	513.5	0.01	0.21	0.79	3.40
BDP2	535.0	5.3	549.0	0.03	0.28	0.95	5.04
BDP3	534.0	7.1	547.0	0.03	0.28	0.93	6.60
BDP4	511.0	10.3	526.0	0.02	0.03 (94%)<br>3.57 (6%)	0.77	7.96
BDP5	511.0	15.2	532.0	0.02	0.02 (97%)<br>3.88 (3%)	0.84	12.79
RB*	556.0	9.8	578.0	0.10	0.56	0.86	8.43
BDP6	675.0	5.2	709.0	0.20	2.04	0.44	2.29
BDP7	675.0	7.9	715.0	0.21	2.05	0.46	3.63
Th*	600.0	3.3	621.0	0.06	0.50	0.79	2.61
C6*	401.0<br>501.0<br>661.0	9.0<br>0.7<br>2.7	669.5	0.18	4.89	0.80	7.20<br>0.56<br>2.16

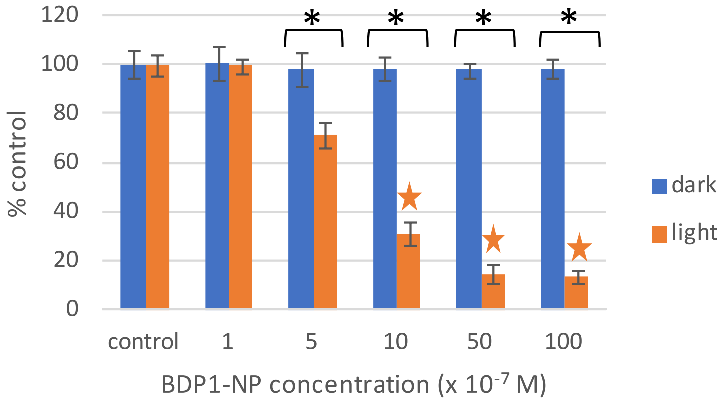

Figure S4. Cell viability (MTT assay) of HeLa cells exposed to the nanosystem BDP1-NP, in dark conditions (blue) and after blue irradiation at 10 J/cm² (red). Stars indicate significant differences with respect to controls. Asterisks indicate significant differences between dark and light conditions at the same concentration.

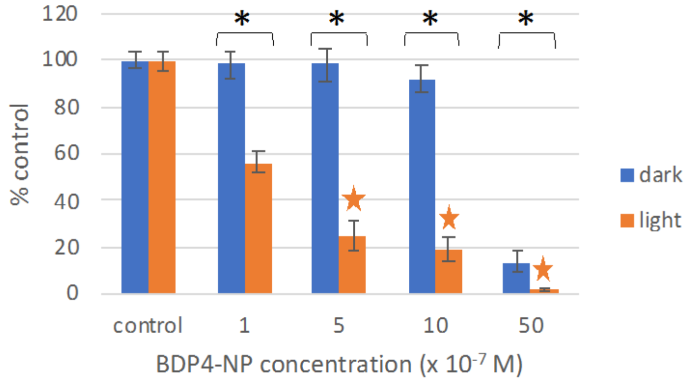

Figure S5. Cell viability (MTT assay) of HeLa cells exposed to the nanosystem BDP4-NP, in dark conditions (blue) and after green irradiation at 10 J/cm² (red). Stars indicate significant differences with respect to controls. Asterisks indicate significant differences between dark and light conditions at the same concentration.

Table S3. EC_{50} in HeLa cells treated with PSs with carboxylic group for 24 h given as PS concentration.

	**Dark (x 10^{-7}M)**	**Light (x 10^{-7}M)**
RB	-	10.40
BDP2	39.2	< 1
BDP4	-	41.90
BDP6	39.8	< 1
C6	81.0	6.85

-: not cytotoxic

Table S4. EC_{50} in HeLa cells treated with PS-NPs for 24 h given as PS concentration.

	**Dark (x 10^{-7}M)**	**Light (x 10^{-7}M)**
RB-PEG-NP-d	-	5.45
BDP1-NP	-	10.07
BDP3-NP	-	4.06
BDP4-NP	33.46	4.43
BDP5-NP	-	1.12
BDP6-NP	-	< 1
C6-NP	-	53.40

-: not cytotoxic

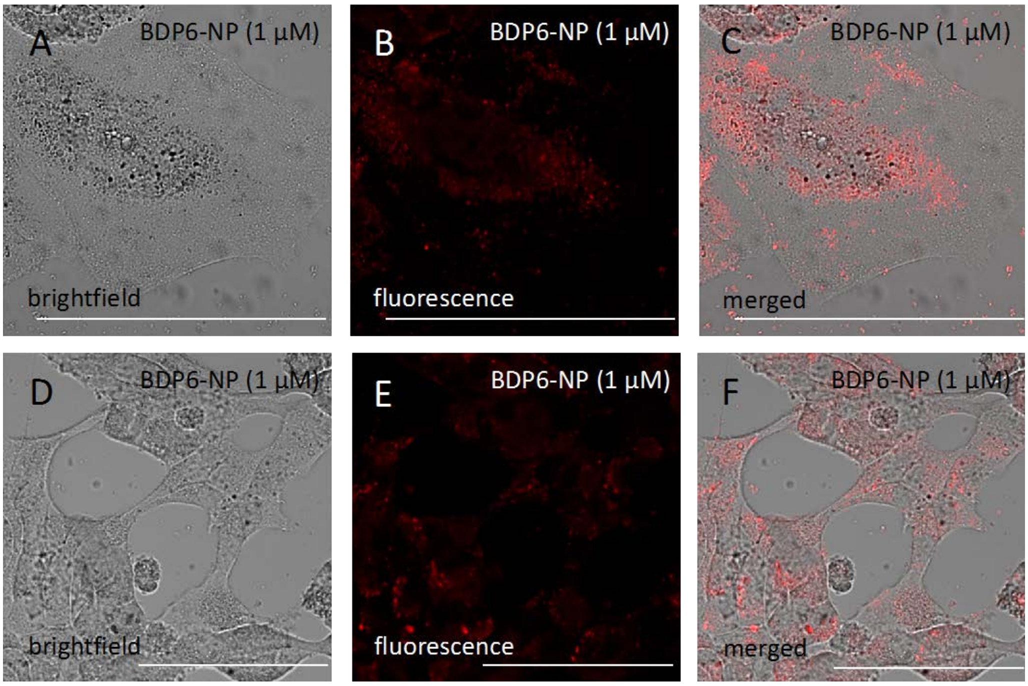

Figure S6. Fluorescence microscopy images (λex = 640 nm and λem = 645-700 nm) of HeLa cells treated with 1 µM BDP6-NP for 24 h. Scale bars = 100 µm.

Synthesis of new BODIPY-based PSs

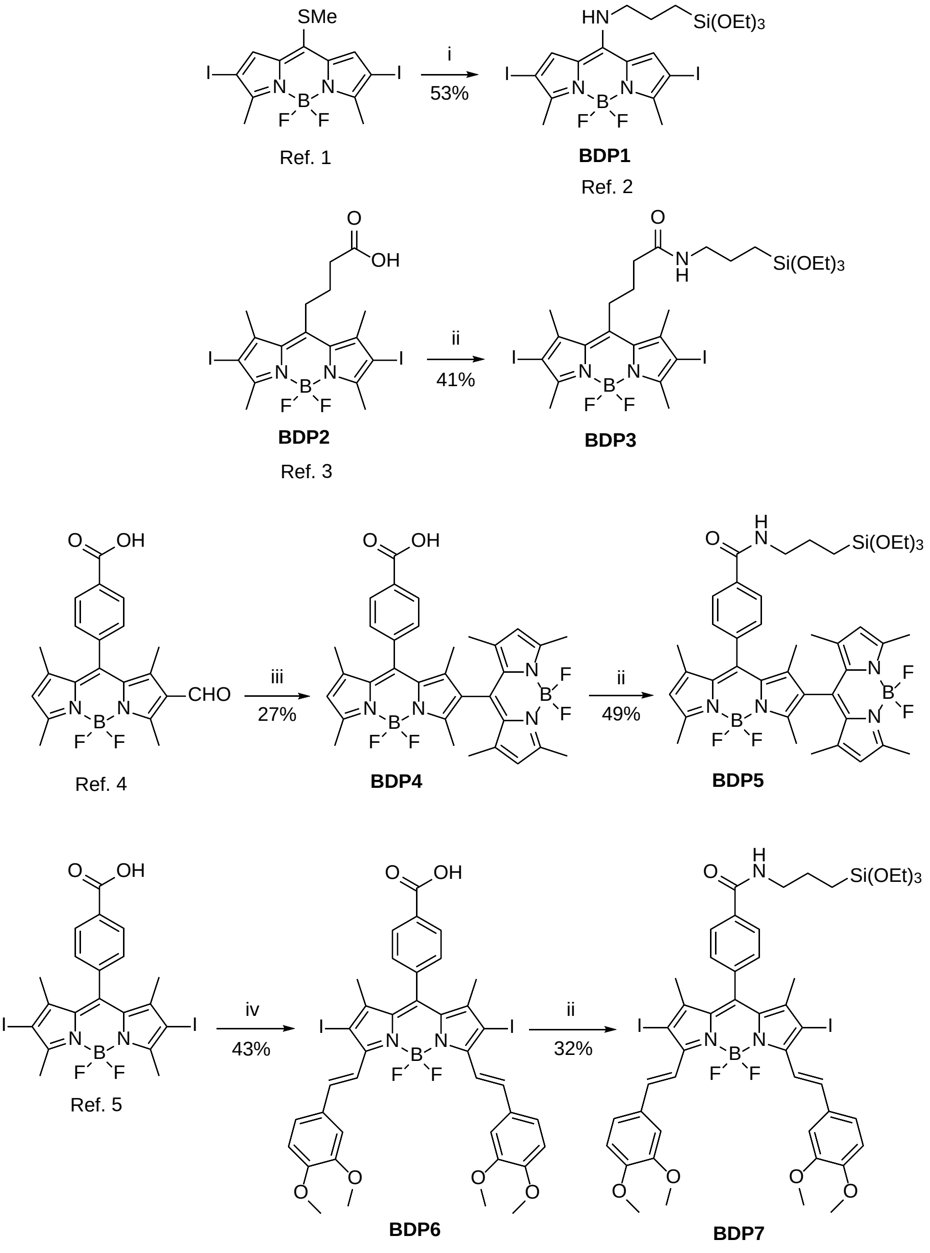

Scheme S1. Synthesis of BODIPYs **BDP1-BDP7**. Reaction conditions: i) NH_2-$(CH_2)_3$-$Si(OEt)_3$, CH_3CN/CH_2Cl_2 (1:1), rt; ii) APTES, TEA, EDC, HOBt, CH_2Cl_2, rt; iii) 2,4-dimethylpyrrole, TFA, DDQ, TEA, BF_3. Et_2O, CH_2Cl_2, rt; iv) 3,4-dimethoxybenzaldehyde, piperidine, AcOH, DMF, 80 ºC, MW [1–5].

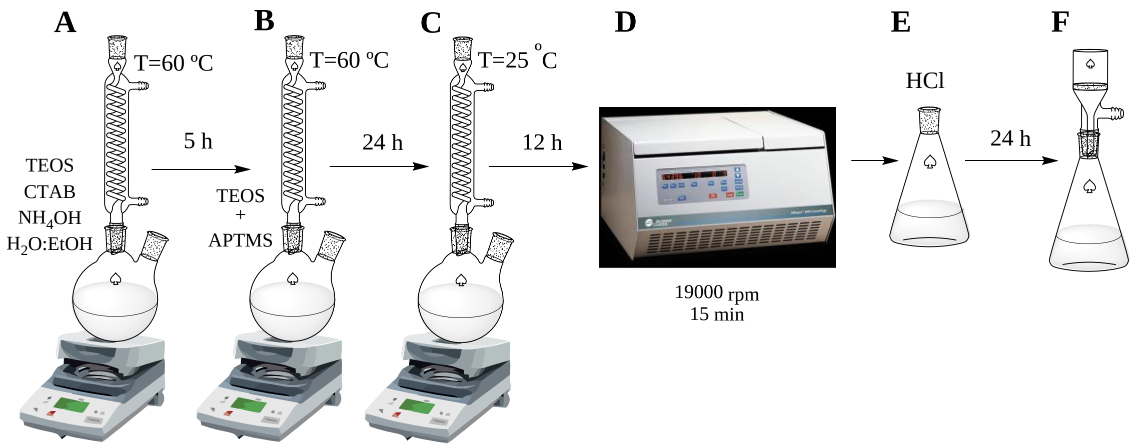

Figure S7. Synthesis of NH-MSN.

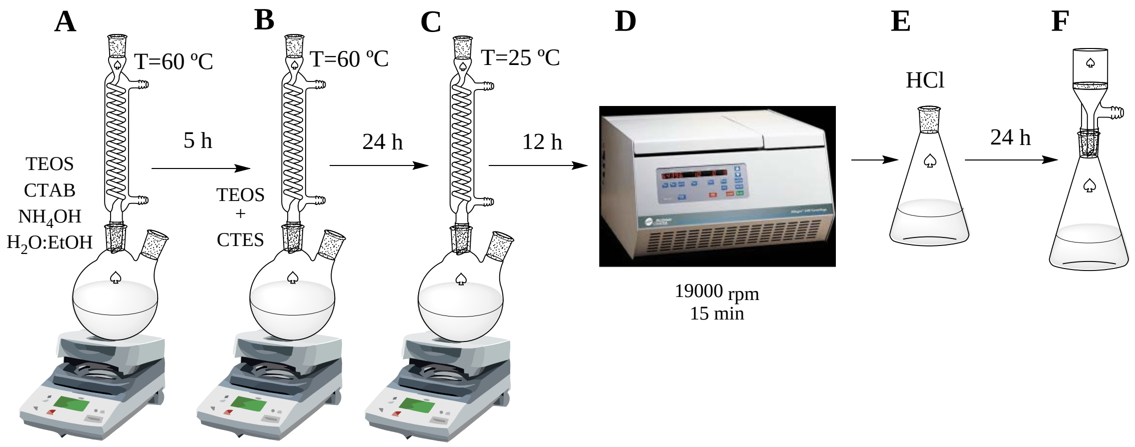

Figure S8. Synthesis of CN-MSN.

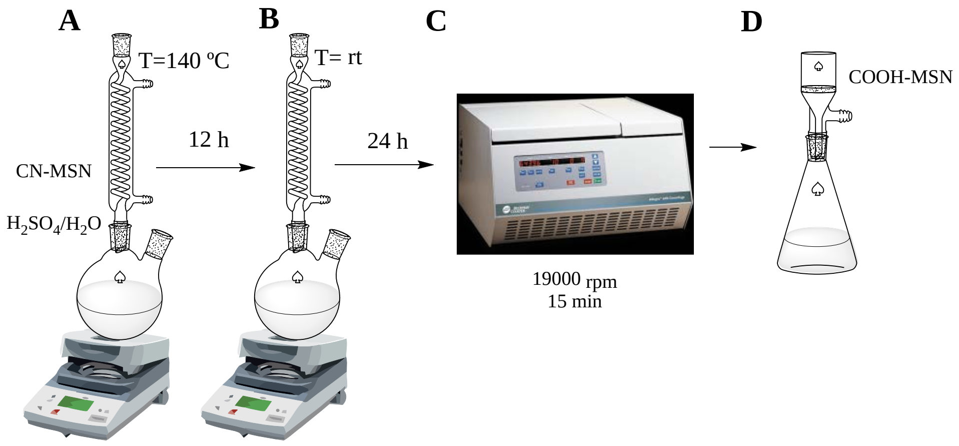

Figure S9. Synthesis of COOH-MSN from CN-MSN.

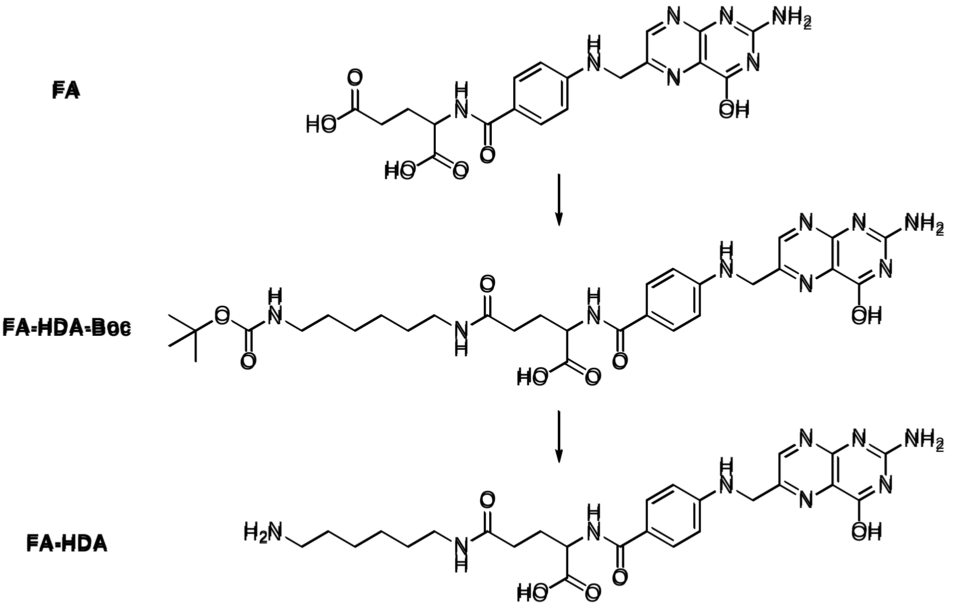

Scheme S2. Folic acid (FA) structure and their derivate FA-HDA-Boc and FA-HDA. **Procedure:** the edged carboxyl group was modified with *N*-Boc-1,6-hexanediamine (Boc-HDA) and the amine groups (FA-HDA) is obtained after removing Boc group, according to the reference.[6]

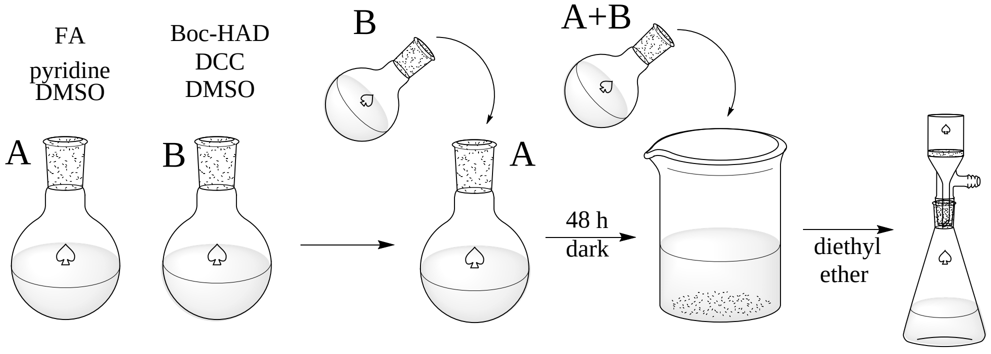

Figure S10. Synthesis of folic acid derivate FA-HDA-Boc.

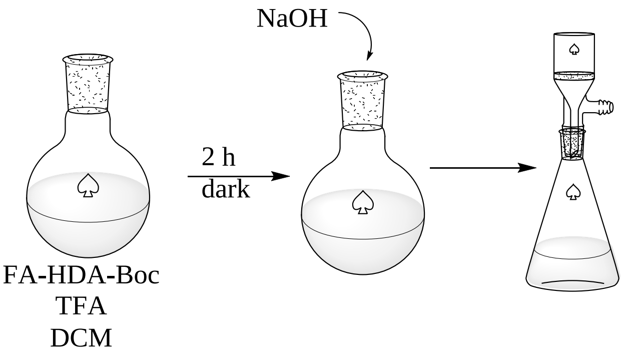

Figure S11. Deprotection of FA-HDA-Boc to obtain FA-HDA.

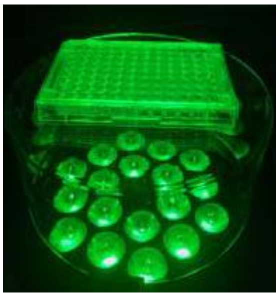

Figure S12. An example of the light irradiation to the HeLa cells by the green light (λ_{ab} 518 nm) devices: LED Par 64 Short Q4-18 (Showtec, Burgebrach, Holland).

^{1}H NMR and ^{13}C NMR spectra

^{1}H NMR (300 MHz, $CDCl_3$) and ^{13}C NMR (75 MHz, $CDCl_3$) spectra of **BDP3**

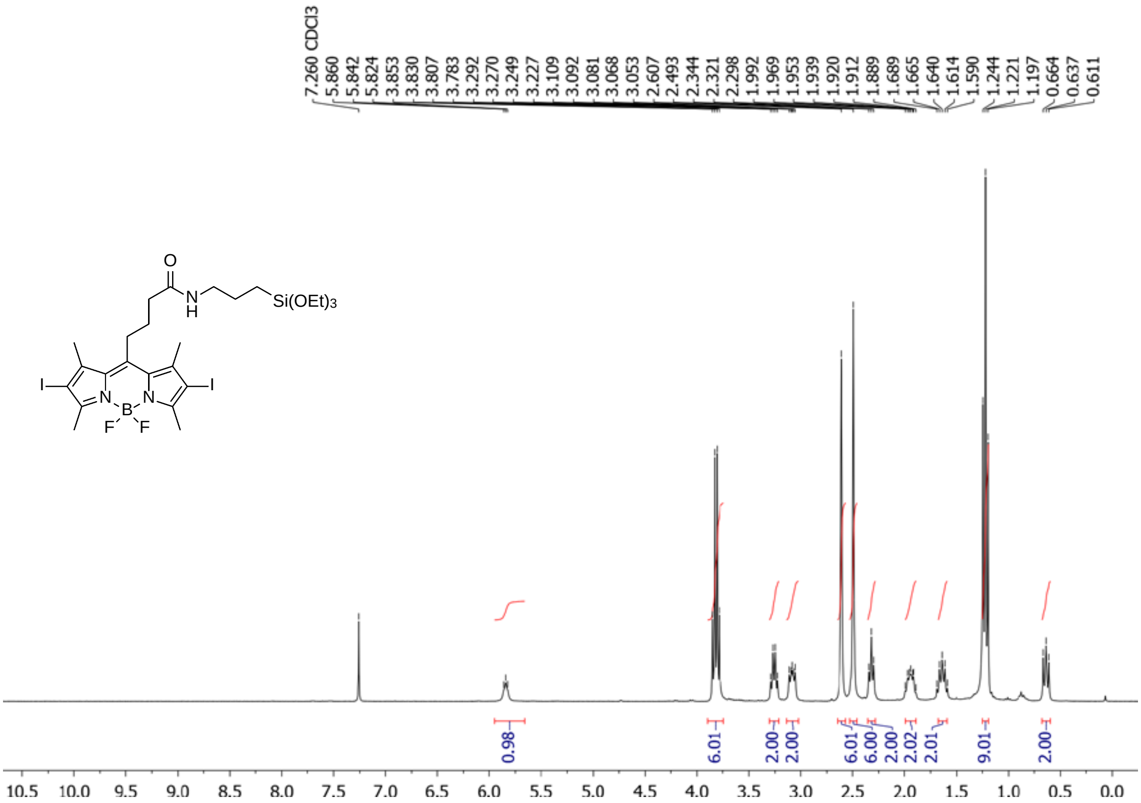

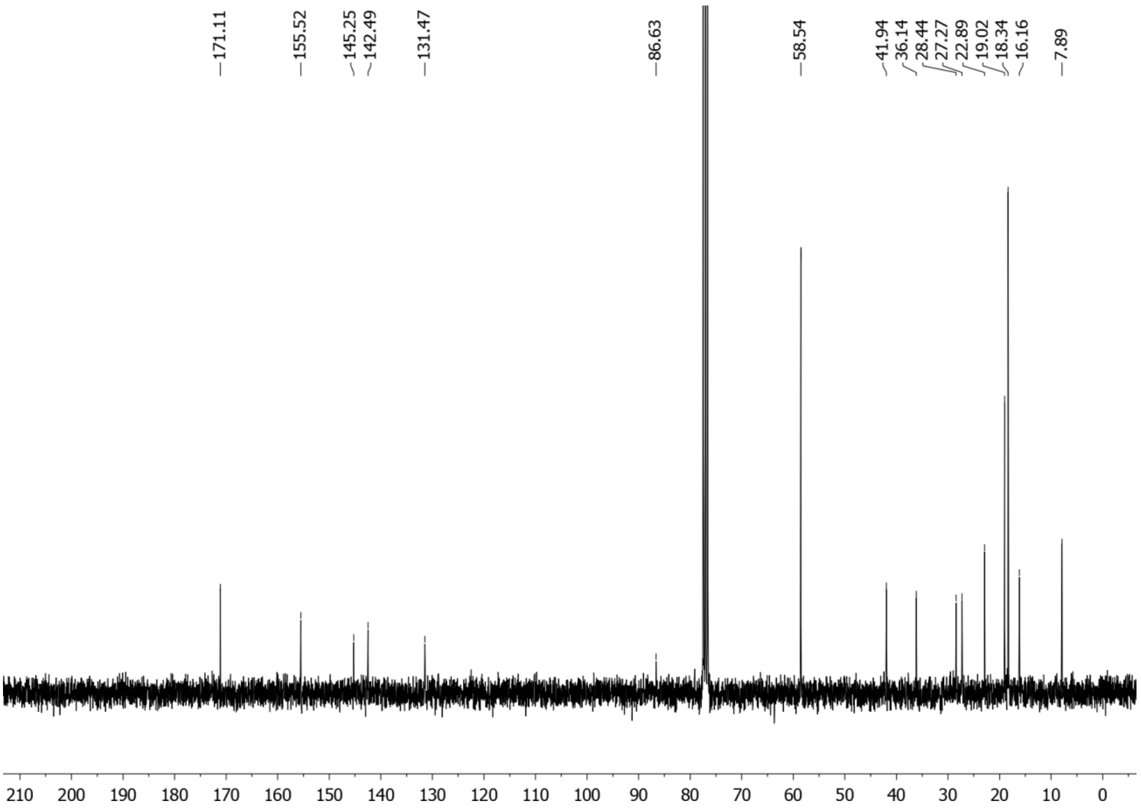

^{1}H NMR (300 MHz, $CDCl_3$) and ^{13}C NMR (75 MHz, $CDCl_3$) spectra of **BDP4**

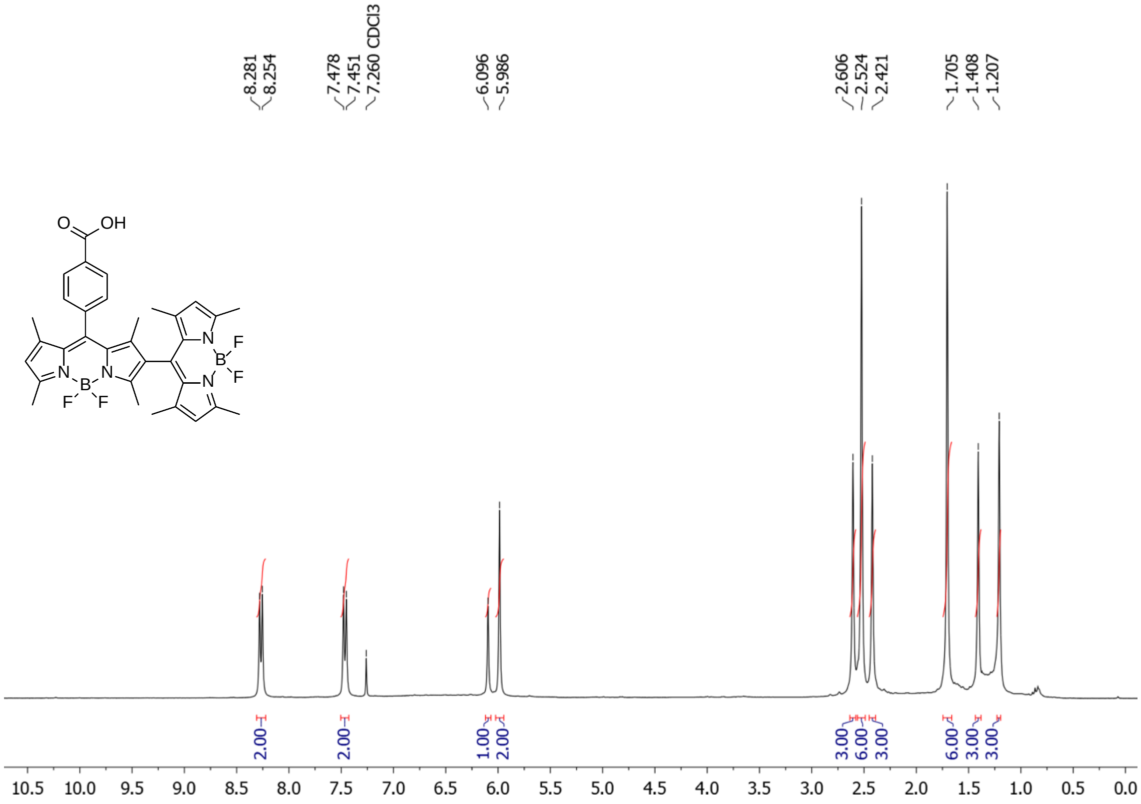

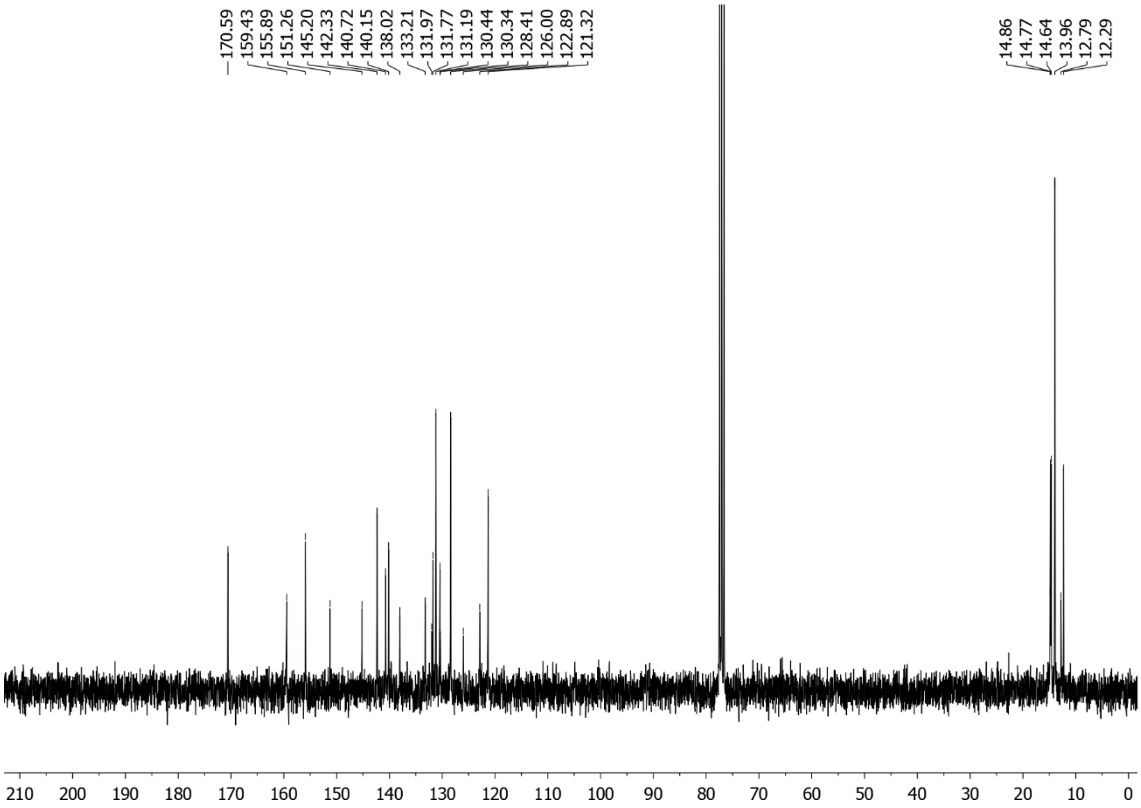

^{1}H NMR (300 MHz, $CDCl_3$) and ^{13}C NMR (75 MHz, $CDCl_3$) spectra of **BDP5**

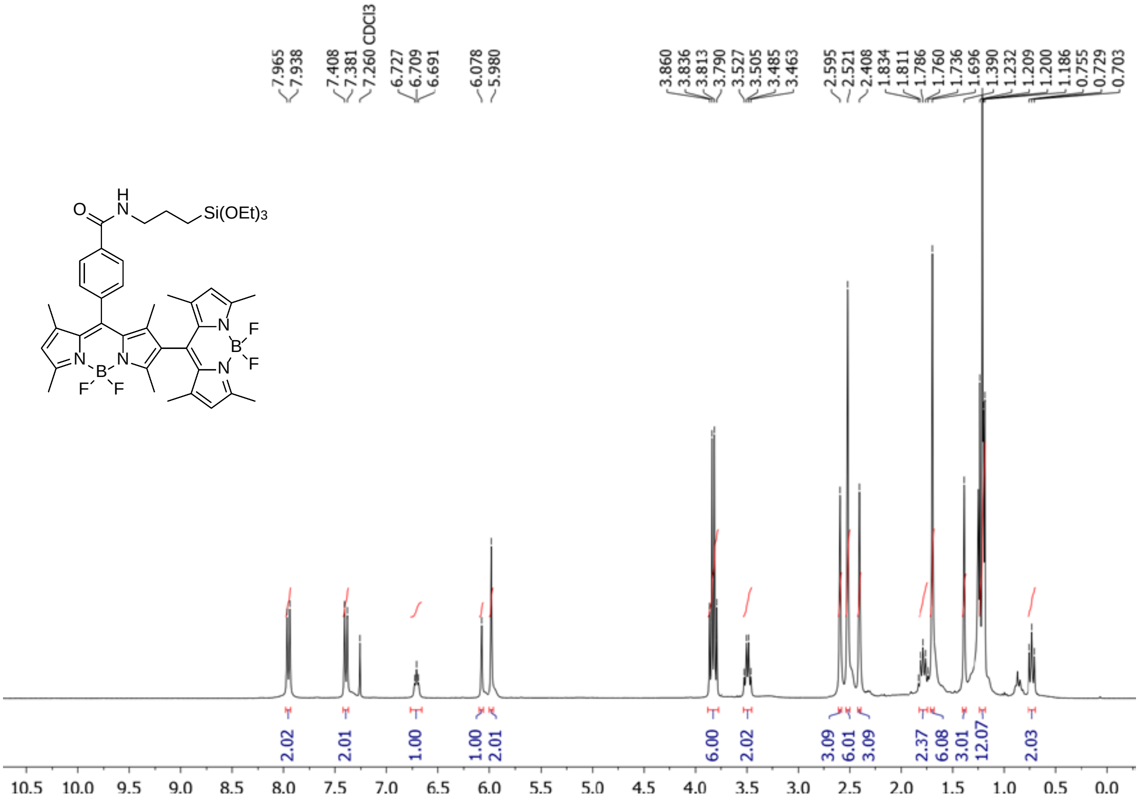

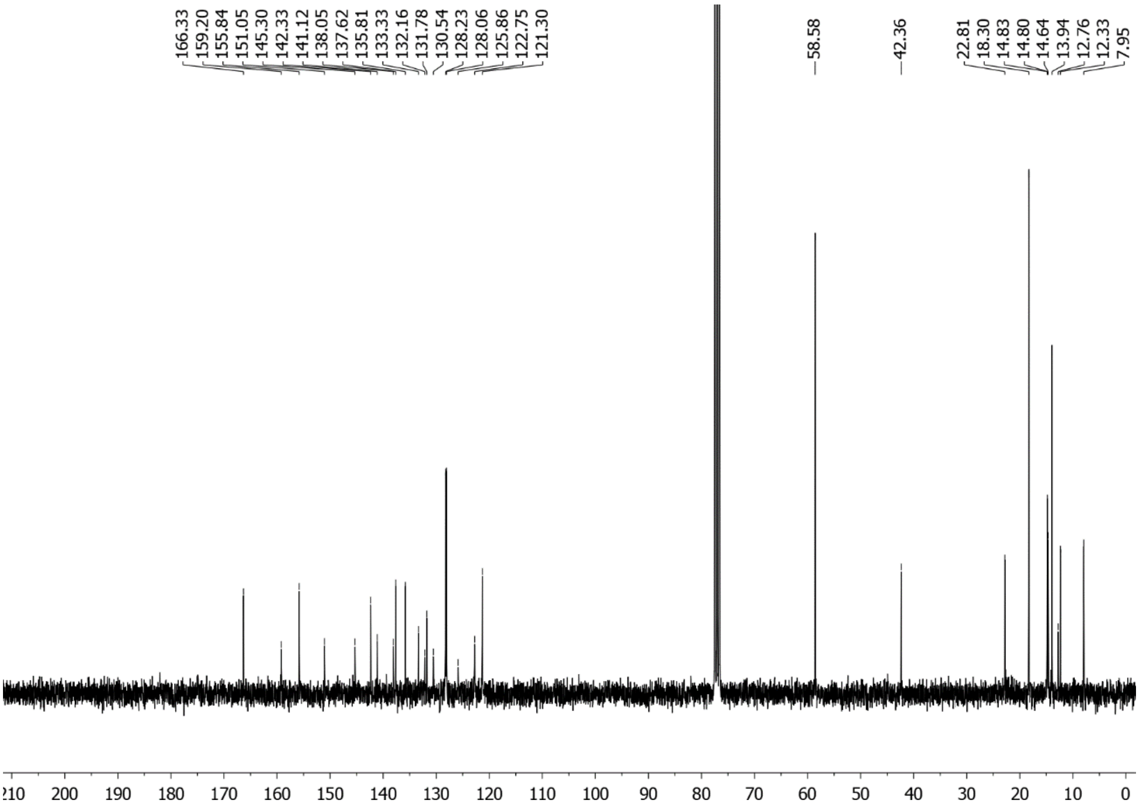

^{1}H NMR (700 MHz, $CDCl_3/CD_3OD$ 4:1) and ^{13}C NMR (176 MHz, $CDCl_3/CD_3OD$ 4:1) spectra of **BDP6**

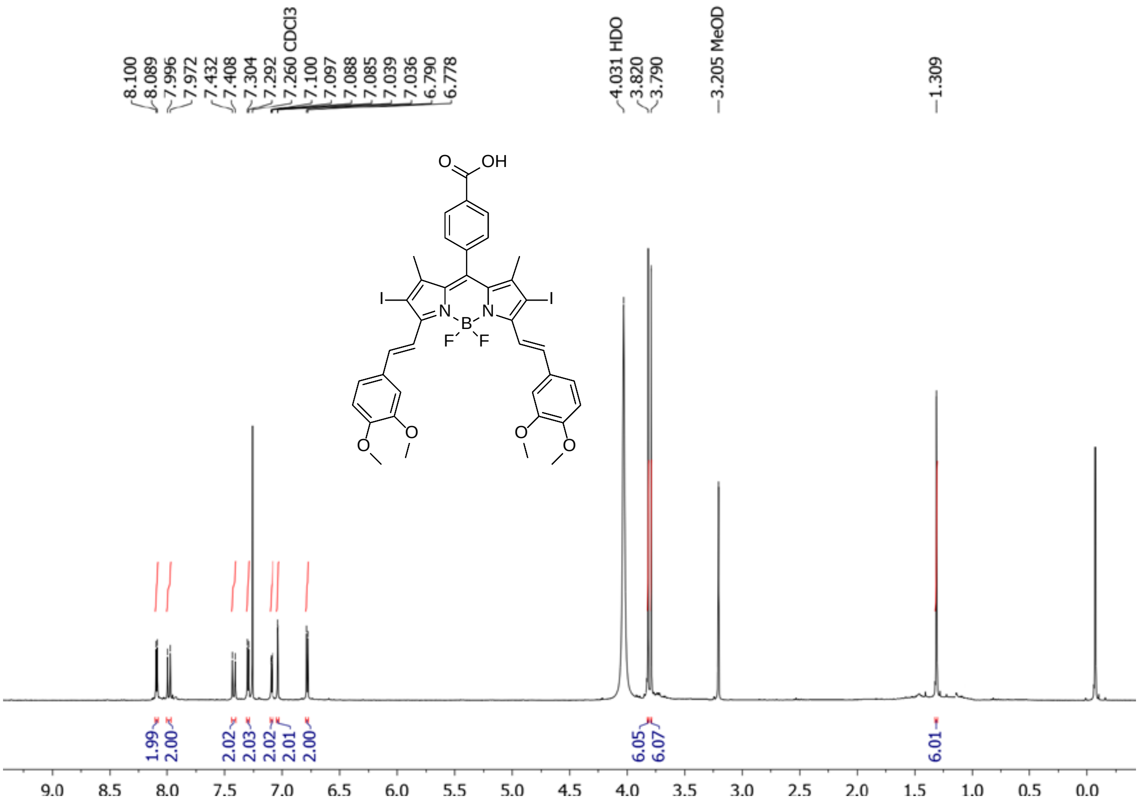

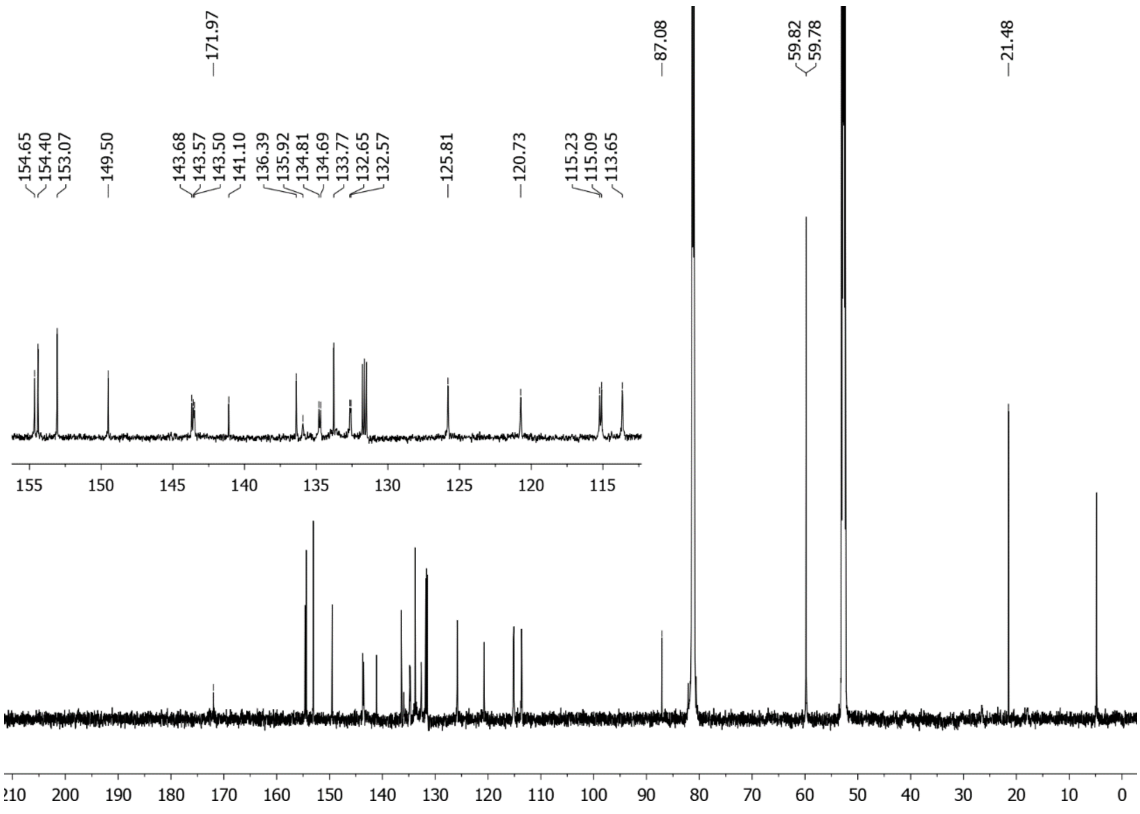

^{1}H NMR (700 MHz, $CDCl_3$) and ^{13}C NMR (176 MHz, $CDCl_3$) spectra of **BDP7**

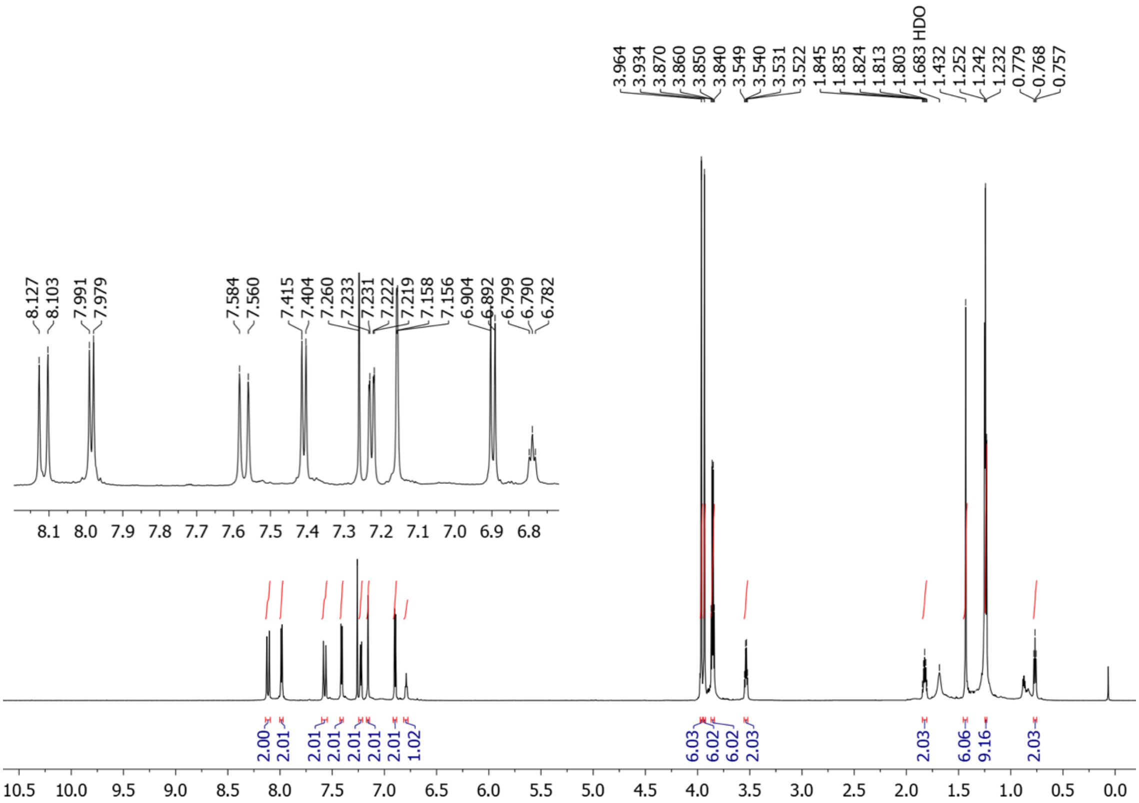

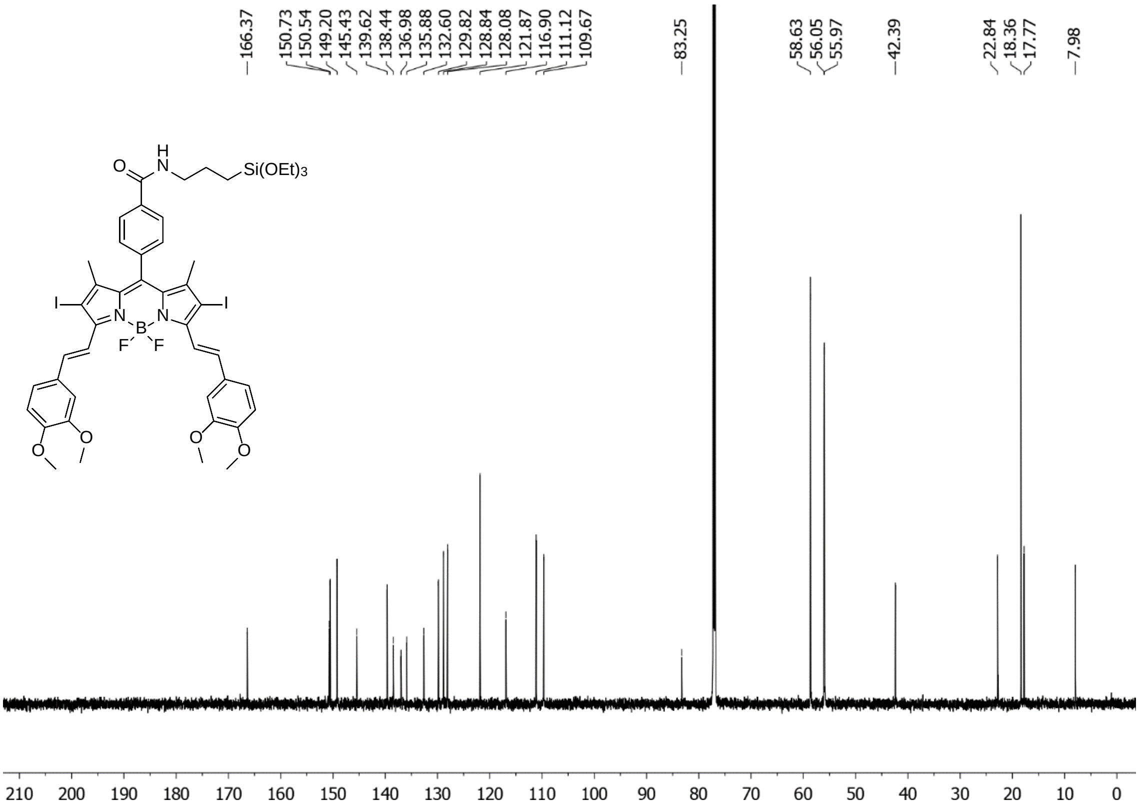

References

1. Gõmez-Durán, C.F.A.; Esnal, I.; Valois-Escamilla, I.; Urías-Benavides, A.; Bañuelos, J.; Lõpez Arbeloa, I.; García-Moreno, I.; Peña-Cabrera, E. Near-IR BODIPY Dyes à la Carte - Programmed Orthogonal Functionalization of Rationally Designed Building Blocks. *Chemistry - A European Journal* **2016**, *22*, 1048–1061.
2. Epelde-Elezcano, N.; Prieto-Montero, R.; Martínez-Martínez, V.; Ortiz, M.J.; Prieto-Castañeda, A.; Peña-Cabrera, E.; Belmonte-Vázquez, J.L.; López-Arbeloa, I.; Brown, R.; Lacombe, S. Adapting BODIPYs to singlet oxygen production on silica nanoparticles. *Physical Chemistry Chemical Physics* **2017**, *19*, 13746–13755.
3. Fraix, A.; Blangetti, M.; Guglielmo, S.; Lazzarato, L.; Marino, N.; Cardile, V.; Graziano, A.C.E.; Manet, I.; Fruttero, R.; Gasco, A.; et al. Light-tunable generation of singlet oxygen and nitric oxide with a bichromophoric molecular hybrid: A bimodal approach to killing cancer cells. *ChemMedChem* **2016**, *11*, 1371–1379.
4. Wu, G.; Zeng, F.; Wu, S. A water-soluble and specific BODIPY-based fluorescent probe for hypochlorite detection and cell imaging. *Analytical Methods* **2013**, *5*, 5589-5596.
5. Guo, S.; Zhang, H.; Huang, L.; Guo, Z.; Xiong, G.; Zhao, J. Porous material-immobilized iodo-Bodipy as an efficient photocatalyst for photoredox catalytic organic reaction to prepare pyrrolo[2,1-a]isoquinoline. *Chemical Communications* **2013**, *49*, 8689-8691.
6. Santiago, A.M.; Ribeiro, T.; Rodrigues, A.S.; Ribeiro, B.; Frade, R.F.M.; Baleizão, C.; Farinha, J.P.S. Multifunctional Hybrid Silica Nanoparticles with a Fluorescent Core and Active Targeting Shell for Fluorescence Imaging Biodiagnostic Applications. *European Journal of Inorganic Chemistry* **2015**, *2015*, 4579–4587.
